# The Association between Socioeconomic Status and Race/Ethnicity with Home Evacuation of Lower Manhattan Residents following the 9/11/2001 World Trade Center Disaster

**DOI:** 10.3390/ijerph21060803

**Published:** 2024-06-19

**Authors:** James E. Cone, Lucie Millien, Cristina Pollari, Jennifer Brite, Heather Badger, John Kubale, Grace Noppert, Sonia Hegde, Robert Brackbill, Mark Farfel

**Affiliations:** 1 New York City Department of Health and Mental Hygiene, World Trade Center Health Registry, New York, NY 11101, USA; 2 York College, City University of New York, New York, NY 11451, USA; 3 Institute for Social Research, University of Michigan, Ann Arbor, MI 48109, USA; 4 Bloomberg School of Public Health, Johns Hopkins University, Baltimore, MD 21205, USA

**Keywords:** World Trade Center disaster, evacuation, socioeconomic status

## Abstract

On 11 September 2001, attacks on the World Trade Center (WTC) killed nearly three thousand people and exposed hundreds of thousands of rescue and recovery workers, passersby, area workers, and residents to varying amounts of dust and smoke. Former New York City Mayor Rudy Giuliani ordered the emergency evacuation of Lower Manhattan below Canal Street, but not all residents evacuated. Previous studies showed that those who did not evacuate had a higher incidence of newly diagnosed asthma. Among the 71,424 who enrolled in the WTC Health Registry in 2003–2004, we evaluated the bivariate association of educational attainment, household income, and race or ethnicity with reported evacuation on or after 9/11/01. We used log binomial regression to assess the relative risks of not evacuating from their home following the 9/11 attacks, adjusting for age, gender, and marital status. Out of a total of 11,871 enrollee residents of Lower Manhattan, 7345 or 61.79% reported evacuating their home on or after 9/11. In a fully adjusted model, the estimated relative risk for not evacuating was elevated for those who identified as non-Hispanic Black, Asian/Pacific Islander, and Hispanic residents compared to non-Hispanic White residents. Residents with a high school diploma/GED had an elevated estimated risk compared to those with at least a bachelor’s degree. Those with lower household incomes had an elevated estimated risk compared to those with the highest income category. These significant inequities will need to be prevented in future disasters.

## 1. Introduction

On 11 September 2001, attacks on the World Trade Center (WTC) killed nearly three thousand people and exposed hundreds of thousands of rescue and recovery workers, passersby, area workers, and residents to varying amounts of dust and smoke, which has been linked to a variety of acute and chronic health conditions including asthma, rhinosinusitis, posttraumatic stress disorder, and gastroesophageal reflux. Carcinogens found in samples of the dust and smoke include asbestos, benzene, polycyclic hydrocarbons, polychlorinated biphenyls, polybrominated diphenyl ethers, dioxins, and furans [1], which have been linked to a variety of adverse health conditions including asthma and cancer. At 11:02 a.m. on 9/11/01, Mayor Giuliani ordered the emergency evacuation of the area south of Canal Street in Lower Manhattan. Over the next several hours, over 1 million people who worked and lived in Lower Manhattan left the area [2]. Boats were used by local mariners to transport between 350,000 and 500,000 people from Lower Manhattan that day, but an unknown number of residents did not evacuate. Previous studies showed that those who did not evacuate had a higher incidence of newly diagnosed asthma and serious psychological distress [3].

The Protection Action Decision Model was developed as a theoretical framework for home evacuation following “natural” disasters, including volcano eruptions, wildland fires, hurricanes, and other weather-related incidents [4]. The decision to evacuate or not is a complex multi-step process, involving prior experience with relief efforts, risk perception [5], fear of theft of household goods [6], social cues of perceiving others preparing to evacuate, levels of community cooperation [7], and weighing warnings and evacuation orders against competing demands. Determining whether evacuating or sheltering in place is the best option largely depends on one’s perception of safety.

Whether the person has access to the means to evacuate (car, bus or train), adequate financial resources, medications, and number and ages of family members or pets may determine what actions are taken [8]. The presence of younger children increases the likelihood of evacuation, while older adults tend to evacuate less often [9]. People who are vulnerable due to poverty, unemployment, and a lower level of education have reduced rates of evacuation.

There is no similar standard theoretical framework for evacuation following terrorist attacks. Furthermore, it is worth noting that the WTC attacks represent one of the few disasters in which those with high socioeconomic status were more likely to be exposed to the immediate effects. In this paper, we examine whether the more socioeconomically disadvantaged were less able to evacuate their residence. Our study questions were as follows: (1) What were the patterns of evacuation immediately after the World Trade Center attacks? (2) What were the socioeconomic factors associated with home evacuation following 11 September 2001; and (3) what were the most common reasons for non-evacuation? 

## 2. Materials and Methods

The World Trade Center Health Registry (WTCHR) was created in 2002 as a longitudinal exposure cohort of persons exposed to the terrorist attack and subsequent collapse of the World Trade Center buildings [3]. The WTCHR was a product of the combined public health efforts of the New York City Department of Health and Mental Hygiene (DOHMH) and the Agency for Toxic Substances and Disease Registry (ATSDR). The WTCHR’s methods have been published previously [3,10]. Briefly, a total of 71,424 persons enrolled in the WTCHR either by phone (95%) or personal (5%) interview in 2003–2004. The enrollment survey included information about demographic factors, physical and mental health-related exposures, symptoms, health conditions, and evacuation details. There have been four subsequent Registry surveys, in 2006-7, 2010-11, 2015-16, and 2020–2021, focused on reported symptoms and acute and chronic health conditions.

### 2.1. Analytic Sample 

The total enrollment in the Registry was 71,424. We first excluded those who were enrolled via proxy (*n* = 3094). Next, we excluded those who did not live south of Canal Street on 9/11/2001 (*n* = 56,225), as this was the dividing line for recruitment eligibility for Lower Manhattan residents. Next, we excluded those who were <18 years of age on 9/11/01 (*n* = 195). Finally, we excluded those who also did not answer the question regarding evacuation in the Wave 1 survey (*n* = 119). The final analytic sample comprised 11,868 enrollees.

### 2.2. Outcomes

A person who evacuated was defined as a Lower Manhattan resident who responded with a “YES” to the question in the Wave 1 survey, “As a result of the World Trade Center disaster, did you have to leave your home?” A person who did not evacuate was defined by a response of “NO” to the same question.

Reasons for not evacuating were defined based on the responses to the WTCHR Wave 2 survey question “If you did not leave your home for at least 24 h between September 11 and September 18, what were some of the reasons (check all that apply)?” Reasons that could be checked included “It wasn’t necessary”, “I wanted to stay with my home”, “I couldn’t afford to leave”, and “I had nowhere else to go”, among others.

### 2.3. Socioeconomic Exposures

Socioeconomic exposures were self-reported at the baseline survey, and included age group on 9/11/01, gender, race/ethnicity, education, income, and marital status.

### 2.4. Ethical Review

This study was approved by the Institutional Review Boards of the NYC DOHMH and the National Institute of Occupational Safety and Health/Centers for Disease Control and Prevention (CDC).

### 2.5. Statistical Analysis

We evaluated the bivariate association of gender, age, educational attainment, household income, marital status, and race/ethnicity with home evacuation using a chi-square test of differences. We fit separate log binomial regression models to assess whether those of varying education, income, or race/ethnicity experienced different estimated relative risks of evacuation from their home following the 9/11 attacks. The models for education and income were adjusted for age, gender, and marital status. We also conducted a descriptive analysis of data from the WTCHR first follow-up survey (Wave 2, 2006–2007) on the reported reasons for not evacuating one’s home on or after 9/11/01.

Analyses were performed using SAS^®^ version 9.4 (SAS Institute, Cary, NC, USA). All statistical tests were two-sided, and statistical significance was indicated if the 95% confidence interval did not contain 1 or if a *p* value was less than 0.05.

## 3. Results

Out of a total of 11,868 enrollee residents of Lower Manhattan, 7345 or 61.9% reported evacuating their home on or after 9/11 while 4523 (39.1%) did not (Table 1). Residents with a high school education/GED were less likely to report evacuation (38%) compared to those with at least a college degree (76%). Similarly, those with a household income of less than USD 25,000 were less likely to report evacuation (41%) compared to those with incomes of USD 150,000 or more (86%). Residents who identified as non-Hispanic Black (50%), Hispanic (46%), Native American (45%), Asian/Pacific Islander (40%), or Other (51%) were less likely to evacuate than those who identified as non-Hispanic White (74%).

In separate fully adjusted models (Table 2), the estimated relative risk (RR) for not evacuating was elevated for those who identified as non-Hispanic Black (RR: 1.69, 95% CI: 1.56–1.84), Asian/Pacific Islander (RR: 2.06, 95% CI: 1.96–2.16), and Hispanic (RR: 1.89, 95% CI: 1.79–2.01) compared to non-Hispanic White residents.

Residents with less than a high school diploma/GED had an elevated estimated risk (RR: 2.90, 95% CI 2.74–3.06) compared to those with at least a bachelor’s degree. Those with a household income of less than USD 25,000 had an elevated estimated risk (RR: 4.46, 95% CI: 3.96–5.03) compared to those with an income of USD 150,000+.

**Figure 1:**
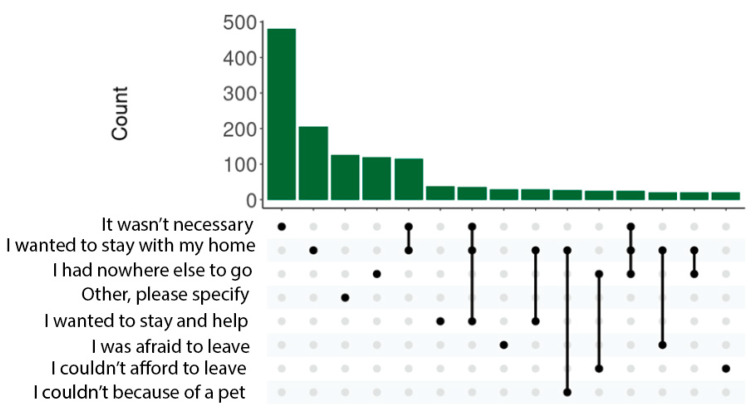
Upset plot for the most common reasons for not evacuating Lower Manhattan after 9/11. The bar plot indicates the frequency of responses among participants (respondents were able to select more than one reason for not evacuating, indicated here by the line plot traversing multiple dots).

The most commonly reported reasons reported for not evacuating were that “it wasn’t necessary”, “I wanted to stay with my home”, and “I had nowhere else to go” (Figure 1).

## 4. Discussion

Although the initial effects of disasters can often affect an entire population regardless of socioeconomic strata, the aftermath disproportionately affects those with the least resources because they are not able to get out of harm’s way, given their physical, financial, and social limitations. Consistent with the findings from Hurricane Katrina [11,12] and other natural disasters [13], WTCHR enrollees who were residents of Lower Manhattan and who reported lower household income and educational attainment were less likely to evacuate. Those who identified as non-Hispanic Black, Asian/Pacific Islander, or Hispanic were also less likely to evacuate.

Our results are still relevant more than 20 years later, given that we will continue to experience natural disasters, e.g., due to climate change or emerging infectious diseases [14]. For example, many residents of New York City left in 2020 during the first wave of the COVID-19 pandemic. Change of address filings with the United States Postal Service documented an increase in move-outs of more than three times the usual numbers, with residents of the wealthiest 10% of neighborhoods (as measured by median income) being 4.6 times as likely to leave than other residents [15].

One study regarding the evacuation of residents prior to Hurricane Katrina showed that income was the strongest and most consistent predictor of pre-storm evacuation [12]. Another study found that evacuation rates prior to the storm differed by race and education: Black residents and those who did not finish high school were less likely to evacuate prior to the storm than white residents and those with a high school education or more [16]. Those with disabilities and older adults also may require special equipment to enable them to evacuate [17].

Important factors likely influencing decisions regarding evacuation include racial and ethnic minority group distrust in health institutions and government agencies [18]. There are also historic legacies of disproportionate impact of disasters on racial and ethnic minorities [19] in addition to discriminatory treatment in the response and recovery following disasters [20]. The intersectionality of race/ethnicity and income likely also plays a role in disaster vulnerability [21,22,23] and needs to be explored further in this context.

This study has several strengths, including the large size of our cohort, longitudinal design, and the fact that we have collected data on the various reasons why people did not evacuate. The results presented in this paper add to the disaster literature as we showed that the more socioeconomically disadvantaged were less likely or able to evacuate their residence despite the fact that they were living in a densely populated urban setting with a relatively high level of overall resources. Limitations include the self-reported nature of our data, lack of data on enrollees’ prior health status, the nature of resources that were available to enable enrollees to evacuate, and study attrition between Waves 1 and 2. The data on the constraints or reasons reported for not evacuating such as desire to stay at their residence, and pets were not robust enough to include in modeling for their potential risk enhancing/reducing effects. Other potential predictors such as distance of residence from the disaster site remain to be addressed by future research.

## 5. Conclusions

Residents of Lower Manhattan who reported lower household income and educational attainment were less likely to evacuate. Those who identified as non-Hispanic Black, Asian/Pacific Islander, or Hispanic were also less likely to evacuate. This suggests that disaster planning and relief needs to more specifically target vulnerable populations in order to avoid exacerbating health disparities in the future.

The following are specific recommendations regarding disaster preparedness for vulnerable populations:

∘ The United States Centers for Disease Control and Prevention and Agency for Toxic Substances and Disease Registry Social Vulnerability Index [https://www.atsdr.cdc.gov/placeandhealth/svi/index.html accessed on 13 June 2024] may be useful to identify populations, particularly in urban areas, needing augmented disaster preparedness outreach including lists of resources to enable timely and effective evacuation should that be necessary. Other measures may be needed in rural areas.
∘ Planning for redundant communication, energy, and transportation networks is important for any area prior to experiencing disasters, but particularly for those without automobiles or other private means of evacuation. Providing subsidized transportation has been proposed as an important means to encourage timely evacuation among low-income populations at risk [13].

## Figures and Tables

**Table 1:** Characteristics of population by evacuation status (Wave 1).

Characteristic at Enrollment	Evacuated Residence (*n*)	Row %	Did Not Evacuate	Row %
Total	7345	61.89	4523	38.11
Age, years				
18–24	803	76.40	248	23.60
25–44	3778	72.95	1401	27.05
45–64	2315	54.50	1933	45.50
≥65	449	32.30	941	67.70
Gender				
Male	3261	62.29	1974	37.71
Female	4084	61.57	2549	38.43
Race/Ethnicity				
Non-Hispanic White	5239	73.73	1867	26.27
Non-Hispanic Black	327	51.33	310	48.67
Hispanic	655	46.75	746	53.25
Asian	979	40.11	1462	59.89
Other/unknown	145	51.24	138	48.76
Education				
<High School	218	17.90	1000	82.10
High School Graduate/GED	563	38.17	912	61.83
Some college	988	56.39	764	43.61
College Graduate or above	5481	76.05	1726	23.95
Household Income (USD)				
<25,000	1097	41.07	1574	58.93
25,000–<50,000	911	51.18	869	48.82
50,000–<75,000	807	61.84	498	38.16
75,000–<150,000	1786	76.39	552	23.61
150,000 or more	1679	86.50	262	13.50
Marital Status				
Now married	3478	62.10	2123	37.90
Not married, living w/partner	730	72.49	277	27.51
Widowed	219	35.78	393	64.22
Divorced	636	57.98	461	42.02
Separated	170	51.99	157	48.01
Never married	2003	67.19	978	32.81

**Table 2:** Adjusted relative risk * for not evacuating from residence in Lower Manhattan according to socio-demographic status. (*n* = 11,868).

Characteristic	ARR	95% CI
Race/Ethnicity *		
Non-Hispanic White	referent	
Non-Hispanic Black	1.69	1.56–1.84
Hispanic	1.90	1.79–2.01
Asian	2.06	1.96–2.16
Other/unknown	1.44	1.18–1.75
Household income at enrollment (USD) *		
<25,000	4.46	3.96–5.03
25,000−<50,000	3.69	3.27–4.18
50,000−<75,000	2.92	2.56–3.34
75,000−<150,000	1.83	1.60–2.08
≥150,000	referent	
Education *		
<High School	2.90	2.74–3.06
High School graduate/GED	2.34	2.20–2.48
Some College	1.78	1.66–1.90
College graduate or above	referent	

* Models were adjusted for gender, age, and marital status.

## Data Availability

World Trade Center Health Registry data may be made available following review of applications to the WTCHR. The data are not publicly available due to privacy or ethical restrictions.
